# 2-Chloro-4,6-bis­(piperidin-1-yl)-1,3,5-triazine

**DOI:** 10.1107/S1600536811000584

**Published:** 2011-01-08

**Authors:** Jasmine P. Vennila, D. John Thiruvadigal, Helen P. Kavitha, G. Chakkaravarthi, V. Manivannan

**Affiliations:** a Department of Physics, Panimalar Institute of Technology, Chennai 602 103, India; b Department of Physics, SRM University, Kattankulathur Campus, Chennai, India; c Department of Chemistry, SRM University, Ramapuram Campus, Chennai 600 089, India; d Department of Physics, CPCL Polytechnic College, Chennai 600 068, India; e Department of Research and Development, PRIST University, Vallam, Thanjavur 613 403, Tamil Nadu, India

## Abstract

The title compound, C_13_H_20_ClN_5_, crystallizes with two mol­ecules in the asymmetric unit. The piperidine rings in both mol­ecules adopt chair conformations. Weak π–π inter­actions [centroid–centroid distance = 3.9815 (8) Å] are observed in the crystal structure.

## Related literature

For the biological activity of related compounds, see: Azev *et al.* (2003 ▶); Steffensen & Simanek (2003 ▶). For bond-length data, see: Allen *et al.* (1987 ▶). For puckering and asymmetry parameters, see: Cremer & Pople (1975 ▶).
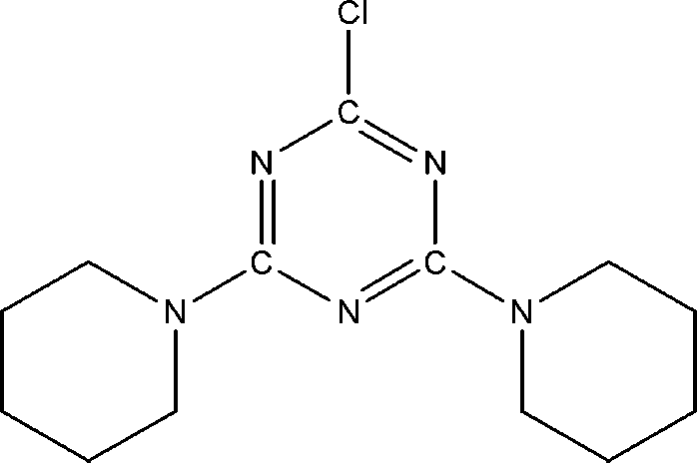


## Experimental

### 

#### Crystal data

C_13_H_20_ClN_5_
*M* *_r_* = 281.79
Triclinic, 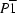

*a* = 10.5085 (2) Å
*b* = 11.7016 (3) Å
*c* = 12.6086 (3) Å
α = 89.813 (1)°
β = 67.967 (2)°
γ = 81.627 (1)°
*V* = 1419.65 (6) Å^3^
*Z* = 4
Mo *K*α radiation
μ = 0.26 mm^−1^
*T* = 295 K
0.22 × 0.18 × 0.16 mm

#### Data collection

Bruker Kappa APEXII diffractometer
Absorption correction: multi-scan (*SADABS*; Sheldrick, 1996 ▶) *T*_min_ = 0.944, *T*_max_ = 0.959
41337 measured reflections
11234 independent reflections
5899 reflections with *I* > 2σ(*I*)
*R*_int_ = 0.030

#### Refinement

*R*[*F* ^2^ > 2σ(*F* ^2^)] = 0.050
*wR*(*F* ^2^) = 0.140
*S* = 1.02
11234 reflections
343 parameters
H-atom parameters constrained
Δρ_max_ = 0.22 e Å^−3^
Δρ_min_ = −0.24 e Å^−3^

### 

Data collection: *APEX2* (Bruker, 2004 ▶); cell refinement: *SAINT* (Bruker, 2004 ▶); data reduction: *SAINT*; program(s) used to solve structure: *SHELXS97* (Sheldrick, 2008 ▶); program(s) used to refine structure: *SHELXL97* (Sheldrick, 2008 ▶); molecular graphics: *PLATON* (Spek, 2009 ▶); software used to prepare material for publication: *SHELXL97*.

## Supplementary Material

Crystal structure: contains datablocks global, I. DOI: 10.1107/S1600536811000584/im2259sup1.cif

Structure factors: contains datablocks I. DOI: 10.1107/S1600536811000584/im2259Isup2.hkl

Additional supplementary materials:  crystallographic information; 3D view; checkCIF report

## Figures and Tables

**Fig. 1.**
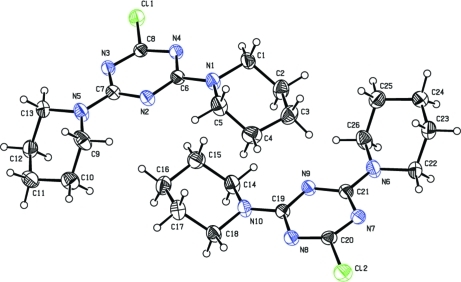
The molecular structure of (I), with atom labels and 30% probability displacement ellipsoids for non-H atoms.

## supplementary crystallographic information

### Comment

1,3,5-Triazine derivatives are of great interest due to their importance as starting materials for drugs and light stabilizers (Azev *et al.*, 2003; Steffensen & Simanek, 2003). In the structure of the title compound (Fig. 1), all bond lengths and angles are in agreement with literature values (Allen *et al.*, 1987).

The piperidine rings N1/C1—C5, N5/C9—C13, N6/C22—C26 and N10/C14—C18 adopt chair conformation [Puckering parameters as defined by Cremer & Pople, 1975: Q = 0.548 (2) Å, θ = 178.3 (2)°, φ = 301 (7)° for the ring N1/C1—C5; Q = 0.555 (2) Å, θ = 179.1 (2)°, φ = 67 (2)° for the ring N5/C9—C13; Q = 0.551 (2) Å, θ = 180.0 (2)°, φ = 161 (3)° for the ring N6/C22—C26; Q = 0.547 (2) Å, θ = 178.7 (2)°, φ = 355 (8)° for the ring N10/C14—C18].

The molecular structure is stabilized by the weak intramolecular C—H···N hydrogen bonds [Table 1] and the crystal structure is stabilized by weak π–π interaction [*Cg*2···*Cg*5 (1 + *x*,*y*,*z*) distance of 3.9815 (8) Å; *Cg*2 and *Cg*5 are the centroids of the rings (N2/C6/N4/C8/N3/C7) and (N7/C20/N8/C19/N9/C21), respectively].

### Experimental

To a stirred solution of piperidine (1 ml) in dichloromethane (10 ml) at 0°C under argon, was added a solution of cyanuric chloride (1.85 g, 10 mmol) in CH_2_Cl_2_ (6 ml) and the reaction mixture was allowed to warm to room temperature. After 14 h, the mixture was partitioned between CH_2_Cl_2 _(20 ml) and saturated aqueous sodium chloride (50 ml). The aqueous phase was extracted with CH_2_Cl_2_ (50 ml), the combined organic layer was dried (MgSO_4_), filtered, evaporated to dryness and purified by recrystallization with ethanol to yield colourless diffraction quality crystals (yield: 68%).

### Refinement

H atoms were positioned geometrically with C—H = 0.97 Å and refined using riding-model approximation with *U*_iso_(H) = 1.2Ueq(C).

### Crystal data

**Table:**

C_13_H_20_ClN_5_	*Z* = 4
*M**_r_* = 281.79	*F*(000) = 600
Triclinic, *P*1	*D*_x_ = 1.318 Mg m^−^^3^
Hall symbol: -P 1	Mo *K*α radiation, λ = 0.71073 Å
*a* = 10.5085 (2) Å	Cell parameters from 9620 reflections
*b* = 11.7016 (3) Å	θ = 2.2–27.3°
*c* = 12.6086 (3) Å	µ = 0.26 mm^−^^1^
α = 89.813 (1)°	*T* = 295 K
β = 67.967 (2)°	Block, colourless
γ = 81.627 (1)°	0.22 × 0.18 × 0.16 mm
*V* = 1419.65 (6) Å^3^	

### Data collection

**Table:**

Bruker Kappa APEXII diffractometer	11234 independent reflections
Radiation source: fine-focus sealed tube	5899 reflections with *I* > 2σ(*I*)
graphite	*R*_int_ = 0.030
ω and φ scans	θ_max_ = 33.8°, θ_min_ = 1.8°
Absorption correction: multi-scan (*SADABS*; Sheldrick, 1996)	*h* = −16→15
*T*_min_ = 0.944, *T*_max_ = 0.959	*k* = −18→18
41337 measured reflections	*l* = −19→19

### Refinement

**Table:**

Refinement on *F*^2^	Primary atom site location: structure-invariant direct methods
Least-squares matrix: full	Secondary atom site location: difference Fourier map
*R*[*F*^2^ > 2σ(*F*^2^)] = 0.050	Hydrogen site location: inferred from neighbouring sites
*wR*(*F*^2^) = 0.140	H-atom parameters constrained
*S* = 1.02	*w* = 1/[σ^2^(*F*_o_^2^) + (0.0539*P*)^2^ + 0.1529*P*] where *P* = (*F*_o_^2^ + 2*F*_c_^2^)/3
11234 reflections	(Δ/σ)_max_ < 0.001
343 parameters	Δρ_max_ = 0.22 e Å^−^^3^
0 restraints	Δρ_min_ = −0.24 e Å^−^^3^

### Fractional atomic coordinates and isotropic or equivalent isotropic displacement parameters (Å^2^)

**Table:**

	*x*	*y*	*z*	*U*_iso_*/*U*_eq_	
Cl1	0.70319 (4)	−0.10443 (3)	0.75663 (3)	0.05514 (11)	
Cl2	−0.24596 (4)	0.58017 (3)	0.75933 (3)	0.06773 (13)	
N1	0.40712 (12)	0.27129 (10)	0.86606 (10)	0.0511 (3)	
N2	0.60248 (11)	0.26026 (9)	0.70082 (9)	0.0445 (3)	
N3	0.75188 (11)	0.07769 (9)	0.64173 (9)	0.0439 (2)	
N4	0.54969 (11)	0.09552 (9)	0.81220 (9)	0.0428 (2)	
N5	0.80109 (13)	0.23811 (11)	0.53713 (10)	0.0570 (3)	
N6	−0.25057 (13)	0.21549 (11)	0.94787 (10)	0.0574 (3)	
N7	−0.24388 (11)	0.38829 (10)	0.85973 (9)	0.0470 (3)	
N8	−0.04785 (11)	0.40643 (9)	0.69089 (9)	0.0460 (3)	
N9	−0.05891 (11)	0.22899 (9)	0.78582 (9)	0.0430 (2)	
N10	0.12982 (12)	0.25155 (9)	0.62450 (10)	0.0492 (3)	
C1	0.31252 (14)	0.22201 (13)	0.96503 (12)	0.0534 (4)	
H1A	0.3136	0.2562	1.0346	0.064*	
H1B	0.3429	0.1394	0.9625	0.064*	
C2	0.16681 (16)	0.24390 (14)	0.96658 (15)	0.0615 (4)	
H2A	0.1040	0.2151	1.0358	0.074*	
H2B	0.1638	0.2024	0.9013	0.074*	
C3	0.11979 (17)	0.37162 (14)	0.96222 (15)	0.0659 (4)	
H3A	0.1112	0.4120	1.0322	0.079*	
H3B	0.0293	0.3832	0.9566	0.079*	
C4	0.22327 (18)	0.42082 (14)	0.85997 (14)	0.0690 (5)	
H4A	0.2216	0.3882	0.7900	0.083*	
H4B	0.1962	0.5039	0.8625	0.083*	
C5	0.36840 (17)	0.39485 (12)	0.85844 (14)	0.0615 (4)	
H5A	0.4330	0.4204	0.7882	0.074*	
H5B	0.3737	0.4369	0.9224	0.074*	
C6	0.52309 (13)	0.20869 (11)	0.79053 (11)	0.0404 (3)	
C7	0.71608 (13)	0.19218 (11)	0.62899 (11)	0.0418 (3)	
C8	0.66359 (12)	0.04159 (10)	0.73336 (11)	0.0388 (3)	
C9	0.77292 (19)	0.35800 (13)	0.51028 (13)	0.0637 (4)	
H9A	0.8475	0.3984	0.5097	0.076*	
H9B	0.6871	0.3960	0.5688	0.076*	
C10	0.76069 (17)	0.36363 (13)	0.39468 (13)	0.0612 (4)	
H10A	0.6788	0.3322	0.3983	0.073*	
H10B	0.7499	0.4437	0.3750	0.073*	
C11	0.88763 (17)	0.29627 (15)	0.30323 (13)	0.0624 (4)	
H11A	0.9677	0.3339	0.2923	0.075*	
H11B	0.8740	0.2952	0.2313	0.075*	
C12	0.91444 (15)	0.17377 (14)	0.33667 (13)	0.0583 (4)	
H12A	0.8394	0.1334	0.3384	0.070*	
H12B	1.0001	0.1336	0.2797	0.070*	
C13	0.92525 (14)	0.17194 (15)	0.45222 (12)	0.0562 (4)	
H13A	0.9362	0.0927	0.4742	0.067*	
H13B	1.0063	0.2048	0.4486	0.067*	
C14	0.18931 (16)	0.12999 (12)	0.62002 (13)	0.0565 (4)	
H14A	0.1827	0.0888	0.5561	0.068*	
H14B	0.1371	0.0954	0.6899	0.068*	
C15	0.33961 (17)	0.11873 (14)	0.60639 (14)	0.0640 (4)	
H15A	0.3450	0.1513	0.6750	0.077*	
H15B	0.3794	0.0375	0.5974	0.077*	
C16	0.42257 (15)	0.17999 (14)	0.50403 (14)	0.0610 (4)	
H16A	0.4271	0.1416	0.4344	0.073*	
H16B	0.5166	0.1767	0.5010	0.073*	
C17	0.35592 (15)	0.30484 (14)	0.51179 (14)	0.0601 (4)	
H17A	0.3621	0.3454	0.5762	0.072*	
H17B	0.4060	0.3415	0.4426	0.072*	
C18	0.20574 (14)	0.31413 (13)	0.52655 (12)	0.0521 (3)	
H18A	0.1638	0.3949	0.5378	0.063*	
H18B	0.1999	0.2826	0.4577	0.063*	
C19	0.00440 (13)	0.29585 (10)	0.70277 (10)	0.0396 (3)	
C20	−0.16935 (14)	0.44079 (11)	0.77202 (11)	0.0440 (3)	
C21	−0.18221 (13)	0.27845 (11)	0.86233 (11)	0.0430 (3)	
C22	−0.38604 (14)	0.25932 (15)	1.03571 (13)	0.0594 (4)	
H22A	−0.4544	0.2140	1.0314	0.071*	
H22B	−0.4147	0.3390	1.0227	0.071*	
C23	−0.37895 (16)	0.25251 (15)	1.15248 (13)	0.0607 (4)	
H23A	−0.4711	0.2758	1.2105	0.073*	
H23B	−0.3196	0.3056	1.1599	0.073*	
C24	−0.32291 (16)	0.13175 (15)	1.17191 (13)	0.0605 (4)	
H24A	−0.3888	0.0805	1.1752	0.073*	
H24B	−0.3116	0.1314	1.2448	0.073*	
C25	−0.18487 (15)	0.08791 (14)	1.07732 (14)	0.0586 (4)	
H25A	−0.1156	0.1326	1.0808	0.070*	
H25B	−0.1553	0.0078	1.0881	0.070*	
C26	−0.19536 (17)	0.09730 (13)	0.96164 (13)	0.0616 (4)	
H26A	−0.1041	0.0749	0.9019	0.074*	
H26B	−0.2558	0.0452	0.9544	0.074*	

### Atomic displacement parameters (Å^2^)

**Table:**

	*U*^11^	*U*^22^	*U*^33^	*U*^12^	*U*^13^	*U*^23^
Cl1	0.0520 (2)	0.04075 (19)	0.0592 (2)	0.00433 (14)	−0.01014 (16)	0.00949 (15)
Cl2	0.0742 (3)	0.0482 (2)	0.0646 (3)	0.01757 (18)	−0.0179 (2)	0.00811 (18)
N1	0.0514 (6)	0.0375 (6)	0.0469 (6)	0.0045 (5)	−0.0031 (5)	0.0045 (5)
N2	0.0512 (6)	0.0386 (6)	0.0376 (6)	−0.0052 (5)	−0.0105 (5)	0.0023 (5)
N3	0.0423 (6)	0.0431 (6)	0.0395 (6)	−0.0031 (5)	−0.0092 (5)	0.0041 (5)
N4	0.0420 (6)	0.0381 (6)	0.0409 (6)	−0.0006 (4)	−0.0095 (5)	0.0057 (5)
N5	0.0596 (7)	0.0488 (7)	0.0466 (7)	−0.0069 (6)	−0.0025 (6)	0.0104 (5)
N6	0.0514 (7)	0.0514 (7)	0.0510 (7)	0.0000 (5)	−0.0017 (6)	0.0114 (6)
N7	0.0456 (6)	0.0464 (6)	0.0432 (6)	0.0032 (5)	−0.0140 (5)	0.0030 (5)
N8	0.0491 (6)	0.0381 (6)	0.0439 (6)	0.0004 (5)	−0.0125 (5)	0.0053 (5)
N9	0.0442 (6)	0.0380 (6)	0.0401 (6)	−0.0026 (5)	−0.0098 (5)	0.0031 (5)
N10	0.0464 (6)	0.0375 (6)	0.0482 (7)	−0.0009 (5)	−0.0023 (5)	0.0069 (5)
C1	0.0510 (8)	0.0489 (8)	0.0439 (8)	0.0047 (6)	−0.0042 (6)	0.0076 (6)
C2	0.0512 (8)	0.0541 (9)	0.0659 (10)	−0.0005 (7)	−0.0100 (7)	−0.0025 (8)
C3	0.0541 (9)	0.0586 (10)	0.0753 (11)	0.0103 (7)	−0.0205 (8)	−0.0064 (8)
C4	0.0862 (12)	0.0459 (9)	0.0638 (10)	0.0182 (8)	−0.0262 (9)	−0.0009 (7)
C5	0.0666 (10)	0.0371 (8)	0.0593 (9)	0.0024 (7)	−0.0034 (8)	0.0018 (7)
C6	0.0438 (6)	0.0370 (6)	0.0369 (6)	−0.0024 (5)	−0.0128 (5)	0.0016 (5)
C7	0.0450 (7)	0.0417 (7)	0.0376 (7)	−0.0086 (5)	−0.0139 (6)	0.0035 (5)
C8	0.0391 (6)	0.0366 (6)	0.0395 (7)	−0.0016 (5)	−0.0150 (5)	0.0024 (5)
C9	0.0852 (11)	0.0450 (8)	0.0488 (9)	−0.0179 (8)	−0.0090 (8)	0.0100 (7)
C10	0.0726 (10)	0.0467 (8)	0.0640 (10)	−0.0146 (7)	−0.0237 (8)	0.0136 (7)
C11	0.0685 (10)	0.0742 (11)	0.0474 (9)	−0.0218 (8)	−0.0213 (8)	0.0113 (8)
C12	0.0466 (8)	0.0683 (10)	0.0529 (9)	−0.0049 (7)	−0.0122 (7)	−0.0012 (7)
C13	0.0401 (7)	0.0713 (10)	0.0491 (8)	−0.0091 (7)	−0.0076 (6)	0.0127 (7)
C14	0.0594 (9)	0.0359 (7)	0.0556 (9)	0.0022 (6)	−0.0043 (7)	0.0030 (6)
C15	0.0658 (10)	0.0557 (9)	0.0587 (9)	0.0145 (7)	−0.0188 (8)	−0.0019 (7)
C16	0.0459 (8)	0.0655 (10)	0.0610 (10)	0.0016 (7)	−0.0120 (7)	−0.0065 (8)
C17	0.0532 (8)	0.0596 (10)	0.0573 (9)	−0.0124 (7)	−0.0080 (7)	−0.0017 (7)
C18	0.0517 (8)	0.0460 (8)	0.0479 (8)	−0.0044 (6)	−0.0079 (6)	0.0086 (6)
C19	0.0429 (6)	0.0342 (6)	0.0394 (7)	−0.0029 (5)	−0.0141 (5)	0.0003 (5)
C20	0.0500 (7)	0.0381 (7)	0.0429 (7)	0.0027 (6)	−0.0198 (6)	0.0007 (5)
C21	0.0440 (7)	0.0445 (7)	0.0391 (7)	−0.0056 (6)	−0.0148 (6)	0.0030 (6)
C22	0.0405 (7)	0.0726 (10)	0.0549 (9)	−0.0043 (7)	−0.0082 (6)	0.0140 (8)
C23	0.0446 (8)	0.0743 (11)	0.0539 (9)	−0.0044 (7)	−0.0098 (7)	−0.0004 (8)
C24	0.0536 (8)	0.0775 (11)	0.0532 (9)	−0.0152 (8)	−0.0215 (7)	0.0146 (8)
C25	0.0494 (8)	0.0528 (9)	0.0723 (10)	−0.0111 (7)	−0.0206 (8)	0.0158 (7)
C26	0.0676 (10)	0.0442 (8)	0.0569 (9)	−0.0082 (7)	−0.0057 (8)	0.0099 (7)

### Geometric parameters (Å, °)

**Table:**

Cl1—C8	1.7493 (12)	C9—H9A	0.9700
Cl2—C20	1.7454 (12)	C9—H9B	0.9700
N1—C6	1.3426 (16)	C10—C11	1.509 (2)
N1—C1	1.4523 (18)	C10—H10A	0.9700
N1—C5	1.4569 (17)	C10—H10B	0.9700
N2—C6	1.3318 (16)	C11—C12	1.510 (2)
N2—C7	1.3403 (16)	C11—H11A	0.9700
N3—C8	1.2964 (16)	C11—H11B	0.9700
N3—C7	1.3637 (16)	C12—C13	1.503 (2)
N4—C8	1.3078 (15)	C12—H12A	0.9700
N4—C6	1.3615 (16)	C12—H12B	0.9700
N5—C7	1.3371 (17)	C13—H13A	0.9700
N5—C13	1.4530 (18)	C13—H13B	0.9700
N5—C9	1.4565 (18)	C14—C15	1.509 (2)
N6—C21	1.3378 (17)	C14—H14A	0.9700
N6—C26	1.4559 (18)	C14—H14B	0.9700
N6—C22	1.4575 (18)	C15—C16	1.507 (2)
N7—C20	1.3037 (17)	C15—H15A	0.9700
N7—C21	1.3587 (16)	C15—H15B	0.9700
N8—C20	1.3082 (17)	C16—C17	1.512 (2)
N8—C19	1.3607 (15)	C16—H16A	0.9700
N9—C19	1.3323 (16)	C16—H16B	0.9700
N9—C21	1.3386 (16)	C17—C18	1.506 (2)
N10—C19	1.3447 (16)	C17—H17A	0.9700
N10—C18	1.4571 (17)	C17—H17B	0.9700
N10—C14	1.4602 (16)	C18—H18A	0.9700
C1—C2	1.508 (2)	C18—H18B	0.9700
C1—H1A	0.9700	C22—C23	1.503 (2)
C1—H1B	0.9700	C22—H22A	0.9700
C2—C3	1.512 (2)	C22—H22B	0.9700
C2—H2A	0.9700	C23—C24	1.506 (2)
C2—H2B	0.9700	C23—H23A	0.9700
C3—C4	1.517 (2)	C23—H23B	0.9700
C3—H3A	0.9700	C24—C25	1.510 (2)
C3—H3B	0.9700	C24—H24A	0.9700
C4—C5	1.504 (2)	C24—H24B	0.9700
C4—H4A	0.9700	C25—C26	1.506 (2)
C4—H4B	0.9700	C25—H25A	0.9700
C5—H5A	0.9700	C25—H25B	0.9700
C5—H5B	0.9700	C26—H26A	0.9700
C9—C10	1.511 (2)	C26—H26B	0.9700
			
C6—N1—C1	122.56 (11)	C13—C12—H12B	109.4
C6—N1—C5	123.04 (11)	C11—C12—H12B	109.4
C1—N1—C5	114.37 (11)	H12A—C12—H12B	108.0
C6—N2—C7	115.16 (11)	N5—C13—C12	110.02 (12)
C8—N3—C7	112.00 (11)	N5—C13—H13A	109.7
C8—N4—C6	111.91 (11)	C12—C13—H13A	109.7
C7—N5—C13	123.28 (12)	N5—C13—H13B	109.7
C7—N5—C9	122.63 (12)	C12—C13—H13B	109.7
C13—N5—C9	114.01 (12)	H13A—C13—H13B	108.2
C21—N6—C26	122.78 (12)	N10—C14—C15	110.52 (13)
C21—N6—C22	123.04 (12)	N10—C14—H14A	109.5
C26—N6—C22	114.18 (12)	C15—C14—H14A	109.5
C20—N7—C21	111.92 (11)	N10—C14—H14B	109.5
C20—N8—C19	111.78 (11)	C15—C14—H14B	109.5
C19—N9—C21	115.25 (11)	H14A—C14—H14B	108.1
C19—N10—C18	122.92 (11)	C16—C15—C14	111.44 (13)
C19—N10—C14	122.06 (11)	C16—C15—H15A	109.3
C18—N10—C14	114.21 (11)	C14—C15—H15A	109.3
N1—C1—C2	110.51 (12)	C16—C15—H15B	109.3
N1—C1—H1A	109.5	C14—C15—H15B	109.3
C2—C1—H1A	109.5	H15A—C15—H15B	108.0
N1—C1—H1B	109.5	C15—C16—C17	110.23 (12)
C2—C1—H1B	109.5	C15—C16—H16A	109.6
H1A—C1—H1B	108.1	C17—C16—H16A	109.6
C1—C2—C3	110.93 (13)	C15—C16—H16B	109.6
C1—C2—H2A	109.5	C17—C16—H16B	109.6
C3—C2—H2A	109.5	H16A—C16—H16B	108.1
C1—C2—H2B	109.5	C18—C17—C16	111.37 (13)
C3—C2—H2B	109.5	C18—C17—H17A	109.4
H2A—C2—H2B	108.0	C16—C17—H17A	109.4
C2—C3—C4	110.40 (12)	C18—C17—H17B	109.4
C2—C3—H3A	109.6	C16—C17—H17B	109.4
C4—C3—H3A	109.6	H17A—C17—H17B	108.0
C2—C3—H3B	109.6	N10—C18—C17	110.78 (12)
C4—C3—H3B	109.6	N10—C18—H18A	109.5
H3A—C3—H3B	108.1	C17—C18—H18A	109.5
C5—C4—C3	111.71 (13)	N10—C18—H18B	109.5
C5—C4—H4A	109.3	C17—C18—H18B	109.5
C3—C4—H4A	109.3	H18A—C18—H18B	108.1
C5—C4—H4B	109.3	N9—C19—N10	117.95 (11)
C3—C4—H4B	109.3	N9—C19—N8	125.01 (11)
H4A—C4—H4B	107.9	N10—C19—N8	117.03 (11)
N1—C5—C4	110.60 (13)	N7—C20—N8	131.13 (12)
N1—C5—H5A	109.5	N7—C20—Cl2	114.49 (10)
C4—C5—H5A	109.5	N8—C20—Cl2	114.38 (10)
N1—C5—H5B	109.5	N6—C21—N9	117.93 (12)
C4—C5—H5B	109.5	N6—C21—N7	117.19 (12)
H5A—C5—H5B	108.1	N9—C21—N7	124.89 (12)
N2—C6—N1	118.59 (11)	N6—C22—C23	110.09 (12)
N2—C6—N4	124.96 (11)	N6—C22—H22A	109.6
N1—C6—N4	116.45 (11)	C23—C22—H22A	109.6
N5—C7—N2	118.54 (12)	N6—C22—H22B	109.6
N5—C7—N3	116.69 (12)	C23—C22—H22B	109.6
N2—C7—N3	124.76 (11)	H22A—C22—H22B	108.2
N3—C8—N4	131.21 (12)	C22—C23—C24	111.25 (13)
N3—C8—Cl1	114.80 (9)	C22—C23—H23A	109.4
N4—C8—Cl1	113.99 (9)	C24—C23—H23A	109.4
N5—C9—C10	110.38 (12)	C22—C23—H23B	109.4
N5—C9—H9A	109.6	C24—C23—H23B	109.4
C10—C9—H9A	109.6	H23A—C23—H23B	108.0
N5—C9—H9B	109.6	C23—C24—C25	111.13 (13)
C10—C9—H9B	109.6	C23—C24—H24A	109.4
H9A—C9—H9B	108.1	C25—C24—H24A	109.4
C11—C10—C9	110.94 (14)	C23—C24—H24B	109.4
C11—C10—H10A	109.5	C25—C24—H24B	109.4
C9—C10—H10A	109.5	H24A—C24—H24B	108.0
C11—C10—H10B	109.5	C26—C25—C24	110.94 (12)
C9—C10—H10B	109.5	C26—C25—H25A	109.5
H10A—C10—H10B	108.0	C24—C25—H25A	109.5
C10—C11—C12	110.62 (13)	C26—C25—H25B	109.5
C10—C11—H11A	109.5	C24—C25—H25B	109.5
C12—C11—H11A	109.5	H25A—C25—H25B	108.0
C10—C11—H11B	109.5	N6—C26—C25	110.16 (13)
C12—C11—H11B	109.5	N6—C26—H26A	109.6
H11A—C11—H11B	108.1	C25—C26—H26A	109.6
C13—C12—C11	111.11 (13)	N6—C26—H26B	109.6
C13—C12—H12A	109.4	C25—C26—H26B	109.6
C11—C12—H12A	109.4	H26A—C26—H26B	108.1
			
C6—N1—C1—C2	−125.43 (14)	C19—N10—C14—C15	−134.48 (14)
C5—N1—C1—C2	56.89 (17)	C18—N10—C14—C15	55.57 (17)
N1—C1—C2—C3	−55.30 (17)	N10—C14—C15—C16	−54.49 (17)
C1—C2—C3—C4	54.33 (18)	C14—C15—C16—C17	54.67 (18)
C2—C3—C4—C5	−53.65 (19)	C15—C16—C17—C18	−54.55 (18)
C6—N1—C5—C4	126.52 (15)	C19—N10—C18—C17	134.52 (14)
C1—N1—C5—C4	−55.82 (17)	C14—N10—C18—C17	−55.63 (17)
C3—C4—C5—N1	53.34 (18)	C16—C17—C18—N10	54.34 (17)
C7—N2—C6—N1	−179.74 (11)	C21—N9—C19—N10	178.84 (11)
C7—N2—C6—N4	−0.14 (19)	C21—N9—C19—N8	−0.91 (19)
C1—N1—C6—N2	178.59 (12)	C18—N10—C19—N9	176.73 (12)
C5—N1—C6—N2	−3.9 (2)	C14—N10—C19—N9	7.66 (19)
C1—N1—C6—N4	−1.0 (2)	C18—N10—C19—N8	−3.49 (19)
C5—N1—C6—N4	176.42 (12)	C14—N10—C19—N8	−172.56 (12)
C8—N4—C6—N2	−0.39 (18)	C20—N8—C19—N9	0.31 (18)
C8—N4—C6—N1	179.22 (11)	C20—N8—C19—N10	−179.44 (11)
C13—N5—C7—N2	−178.52 (12)	C21—N7—C20—N8	−1.7 (2)
C9—N5—C7—N2	−1.9 (2)	C21—N7—C20—Cl2	177.15 (9)
C13—N5—C7—N3	0.9 (2)	C19—N8—C20—N7	1.2 (2)
C9—N5—C7—N3	177.51 (13)	C19—N8—C20—Cl2	−177.71 (9)
C6—N2—C7—N5	−179.76 (12)	C26—N6—C21—N9	1.0 (2)
C6—N2—C7—N3	0.87 (19)	C22—N6—C21—N9	−179.42 (13)
C8—N3—C7—N5	179.68 (11)	C26—N6—C21—N7	−179.10 (13)
C8—N3—C7—N2	−0.93 (18)	C22—N6—C21—N7	0.5 (2)
C7—N3—C8—N4	0.3 (2)	C19—N9—C21—N6	−179.88 (12)
C7—N3—C8—Cl1	−179.40 (9)	C19—N9—C21—N7	0.25 (19)
C6—N4—C8—N3	0.3 (2)	C20—N7—C21—N6	−178.97 (12)
C6—N4—C8—Cl1	−179.99 (8)	C20—N7—C21—N9	0.90 (19)
C7—N5—C9—C10	−119.80 (16)	C21—N6—C22—C23	−122.32 (16)
C13—N5—C9—C10	57.09 (18)	C26—N6—C22—C23	57.27 (18)
N5—C9—C10—C11	−54.29 (18)	N6—C22—C23—C24	−54.38 (18)
C9—C10—C11—C12	54.06 (17)	C22—C23—C24—C25	53.98 (17)
C10—C11—C12—C13	−54.84 (17)	C23—C24—C25—C26	−53.93 (18)
C7—N5—C13—C12	119.29 (15)	C21—N6—C26—C25	122.17 (15)
C9—N5—C13—C12	−57.58 (18)	C22—N6—C26—C25	−57.43 (18)
C11—C12—C13—N5	55.47 (17)	C24—C25—C26—N6	54.50 (17)

### Hydrogen-bond geometry (Å, °)

**Table:**

*D*—H···*A*	*D*—H	H···*A*	*D*···*A*	*D*—H···*A*
C9—H9B···N2	0.97	2.32	2.7637 (18)	107
C13—H13A···N3	0.97	2.30	2.7472 (17)	107
C14—H14B···N9	0.97	2.31	2.7497 (18)	106
C18—H18A···N8	0.97	2.32	2.7560 (18)	106
C22—H22B···N7	0.97	2.30	2.7532 (19)	107
C26—H26A···N9	0.97	2.31	2.7534 (18)	107
